# Duration of exclusive breastfeeding; validity of retrospective assessment at nine months of age

**DOI:** 10.1186/1471-2431-11-80

**Published:** 2011-09-14

**Authors:** Suneth B Agampodi, Suranga Fernando, Samath D Dharmaratne, Thilini C Agampodi

**Affiliations:** 1 Department of Community Medicine, Faculty of Medicine and Allied Sciences, Rajarata University of Sri Lanka. Saliyapura, Sri Lanka; 2 MOH Office, Naula, Sri Lanka; 3 Department of Community Medicine, Faculty of Medicine, University of Peradeniya, Peradeniya, Sri Lanka

## Abstract

**Background:**

In cross sectional, case control and retrospective cohort studies, duration of Exclusive Breastfeeding (EBF) usually depends on maternal recall. Retrospective data are often subjected to recall bias and could lead to a potential for exposure misclassification. The purpose of the present paper is to assess the validity of maternal recall of EBF duration during infancy, after cessation of EBF and to evaluate the two methods to collect retrospective data on EBF.

**Methods:**

A cohort study was carried out in Naula Medical Officer of Health (MOH) area. Study cohort included all infants born during the months of February to April 2008 and currently residing in Naula MOH area. Baseline data collection was carried out using the pregnancy record, the child health development record and by using an interviewer administered structured questionnaire. Data extraction from the pregnancy record and the child health development record were carried out by public health midwives. The interviewer administered structured questionnaire was administered by the MOH during the follow-up visits. Duration of EBF was assessed in three ways; based on prospective data since birth: Retrospective data based on an event calendar: and the Mother reported EBF duration.

**Results:**

A total of 114 mother-infant pairs were recruited and followed up. Proportion of infants receiving EBF up to the completion of the sixth month by the three methods were; data since birth (actual EBF rate) - 23.9%; mother reported data - 77.7% and event calendar method - 41.3%. Median duration of EBF reported in the three methods was 5, 6, and 5 respectively. A statistically significant difference was observed in these differences from Kaplan-Meire Survival analysis (Log rank test - Chi square-63.4, p < 0.001). Validity of retrospective methods was analysed using data since birth as the gold standard. Sensitivity of both methods to detect exclusively breastfed babies were 100.0%. Specificity of mother recall data was 26.2% (95%CI-17.9, 36.8%) compared to 75.0% (95% CI-64.5, 83.2%) in the event calendar method.

**Conclusions:**

Retrospective evaluation methods systematically overestimate the duration of EBF. Maternal recall data provide highly unspecific data whereas use of an event calendar provided more valid data. Reporting of data accrual methods in breastfeeding studies will allow the readers to interpret findings accurately and the use of event calendars rather than direct questioning as a valid method of determining EBF is recommended.

## Background

Breastfeeding is one of the most cost effective interventions for reducing the global burden of childhood mortality and morbidity. More than 13% of under five deaths can be averted globally each year by promoting exclusive breastfeeding [1]. Studies on breastfeeding continue to play a major role in world literature due to its importance in promoting child health.

Breastfeeding recommendations are subjected to change with the accumulation of new evidence. Global health authorities recommend exclusive breastfeeding up to the completion of the sixth month [2,3]. Studies on the effects of exclusive breastfeeding depend heavily upon the definitions used and the data accrual methods. For cross sectional studies, among infants less than 6 months of age, World Health Organization (WHO) recommends the 24-hour recall method to assess the breastfeeding situation in communities [4] whereas some authors challenge the validity of this WHO recommended method compared to exclusive breastfeeding since birth [5-7] method.

When the age of the child is more than six months, there are no consensuses about the data accrual method to obtain the duration of EBF. Further, duration of EBF in a community could be ideally determined only after the cessation of EBF (after six months). Hence, in cross sectional, case control and retrospective cohort studies on childhood illnesses where breastfeeding is a major determinant, duration of EBF is usually determined based on maternal recall. This retrospective evaluation is often used because it is much more efficient than prospective studies for this purpose. Retrospective data are often subjected to recall bias which could lead to a potential for exposure misclassification thus resulting in biased measures of association. Previously, several studies elsewhere have shown the problem of reliability of maternal recall data on estimating the duration of EBF [8-12]. Previously, we showed the low validity of the 24-hour recall data as a method of measuring current status of breastfeeding [5]. The purpose of the present paper is to assess the validity of maternal recall of EBF duration at nine months and to evaluate the validity of two methods to collect retrospective data to estimate the duration of EBF.

## Methods

The present study was carried out from February 2008 to April 2009 in Naula Medical Officer of Health (MOH) area in the Matale district of the Central Sri Lanka. Data for this study was obtained from a cohort study conducted in Naula (Naula breastfeeding cohort). All infants born during the months of February to April 2008 and residing in Naula MOH area at the time of data collection were the study population. All eligible mother-infant pairs were selected from the Birth and Immunization (BI) registers of Public Health Midwives (PHM). Infants registered in BI registers, but planning to move out from the area before completing the first year of life were excluded from the study. PHMs explained the study to care givers and informed verbal consent was obtained prior to data collection. Baseline data collection was carried out using the pregnancy record, the child health development record and by using an interviewer administered structured questionnaire. All infants were followed up by PHMs monthly at weighing clinics, starting from the first month after birth. MOH Naula followed this cohort during routing immunization clinics at completion of 2^nd^, 4^th^, 6^th^, 9^th ^and 12^th ^months after birth. Data extraction from pregnancy records and child health development records were carried out by PHMs. Interviewer administered structured questionnaire was administered by the MOH during the follow-up visits.

The EBF was defined according to the WHO recommendation; "Infant should receive only breast milk from his/her mother or wet nurse or expressed breast milk and no other liquid or solids with exception of drops or syrups consisting of vitamins mineral supplements or medicines"

Duration of exclusive breastfeeding was assessed in the following ways.

Firstly based on data since birth: this definition was based on the prospective assessment of breastfeeding status obtained during follow-up visits. Feeding practices was assessed in each follow-up visit by the MOH during the first six months and the duration of exclusive breastfeeding was determined using an interviewer administered structured questionnaire. The questionnaire included a list of locally prevalent breast milk supplements, complementary feeds, and water based infant foods/drinks used in Matale area. This list included 13 major items categorized in groups including water. Use of food/liquid items in the list, date of introduction of specific food items, frequency of the use and the quantity were also recorded (an event calendar). Recall period for the follow-up was two months at each visit. During monthly follow-ups PHM assessed the feeding status verbally and reported any significant finding to the MOH as a supplement to two months recall period. Once a mother reported that they introduced an item from the list or other food or liquid item, duration of exclusive breastfeeding was calculated to the date of introduction of the specific food item and data collection with regard to EBF duration using data since birth was discontinued.

Secondly using retrospective data based on an event calendar: EBF was retrospectively evaluated after completion of the ninth month using the event calendar method described above. In this evaluation respondents were asked whether they have provided the items indicated in the list, if so the date of introduction of the food item and the frequency of administration and quantity.

Lastly, using the Mother reported EBF duration: After completion of the 9^th ^month, respondents were asked about the duration of EBF using one single question (at what age did you discontinue EBF?).

The main outcome variables were the proportion of infant's breastfed exclusively at the completion of sixth months, and the duration of EBF. Data analysis was carried out to evaluate the validity of reported duration of EBF data. Validity of each method to determine the proportion of mothers practising EBF for six months was evaluated using sensitivity and specificity, considering data since birth as the gold standard.

Ethical clearance for the study was obtained from Ethical review committee, Faculty of Medicine, University of Peradeniya. Administrative clearance for the study was obtained from the Medical officer of health, Naula.

## Results

A total of 114 mother-infant pairs were recruited and were followed up. The mean age of the mothers was 27 years and standard deviation 5.3 years. Only six mothers were employed while the remaining 108 mothers were housewives. All 114 mothers had completed primary education. However, only one mother had studied beyond secondary education. The mean birth weight of infants was 2.85 kg (SD 0.47 kg). Study sample consisted of 61 (53.5%) male and 53 (46.5%) female infants. Five (4.4%) infants were reported as premature (< 37 weeks) deliveries and two (1.8%) were admitted to the Special Care Baby Unit (SBU) immediately after birth. First-born babies accounted for 49.1% (n = 56) of the study sample.

According to baseline data, the breastfeeding initiation rate was 100% at the time of discharge and 109 (95.6%) infants received the first feed within an hour of delivery. All 114 mother-infant pairs were followed up for 6 months. From sixth to ninth months, 11 participants were lost to follow-up and only 103 were available for the final analysis.

As evaluated by data since birth, obtained during prospective follow-up visits, only 23.9% (n = 27) practiced EBF up to the completion of the sixth month. At this age, 41 (36%) has started complementary feeding and 45 (39.5%) were practising predominant breastfeeding. The median duration of exclusive breastfeeding was 5 months with an inter-quartile range of 4 to 5 months. Of the 87 mothers who discontinued EBF before completion of the sixth month, 60 (52.6%) gave water as the first food. However, 21 (18.4%) mothers gave only water in addition to breast milk till the completion of the sixth month.

**Figure 1:**
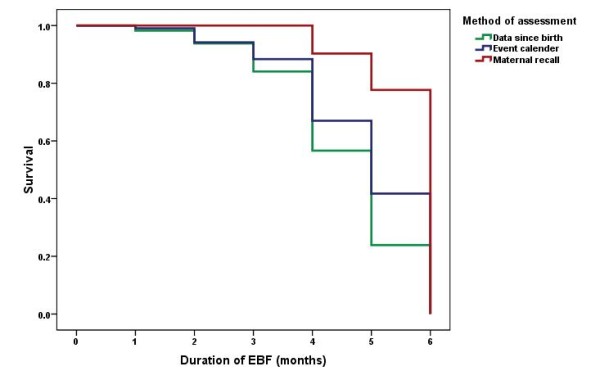
**Survival curves of duration of exclusive breastfeeding by assessment method**.

At completion of nine months, 77.7% were reported as exclusively breastfed for six months and the median duration of EBF was six months. Based on the data collected through the event calendar method, at the completion of the ninth months, this proportion was 41.3% and the median duration was five months. Duration of EBF reported in maternal recall data and event calendar data were compared with the data since birth using Kaplan-Meire survival analysis (please see Figure 1). It showed statistically significant differences in the three survival curves representing the three methods of data collection on duration of EBF (Log rank test - Chi square-63.4, p < 0.001)

Mother reported data and data collected through event calendar method was validated against data since birth to evaluate the ability of the above two methods to estimate EBF for six months. Table 1 shows the distribution of exclusively breastfed and non-exclusively breastfed babies at completion of sixth month.

**Table 1:** Comparison of event calendar and maternal recall data on 6 months EBF with data since birth

	Event calendar			Mother reported EBF		
Data since birth	EBF	N-EBF	Total	EBF	N-EBF	Total
EBF	23	0	23	23	0	23
N-EBF	20	60	60	59	21	80
Total	43	60	103	82	21	103

EBF- Exclusive Breast Feeding, N-EBF- non - exclusive breast feeding

Test parameters were calculated to assess sensitivity, specificity, and predictive values. Each method correctly identified all those infants received only breast milk during first six months of life (sensitivity-100%). However, specificity varied widely. According to the maternal recall method EBF was only 26.2% while in the event calendar method it was 64.5%. Table 2 shows the sensitivity, specificity and predictive values of the two methods.

**Table 2:** Test parameters of event calendar method and maternal recall method, in comparison to data since birth to correctly identified EBF for six months

	Event calendar EBF		Mother reported EBF	
	Estimate	95% CI	Estimate	95% CI
Sensitivity	100.0	85.7, 100	100.0	85.69, 100.0
Specificity	75.0	64.5, 83.2	26.2	17.86, 36.82
Positive Predictive Value	53.5	38.9, 67.5	28.1	19.48, 38.59
Negative Predictive Value	100.0	90.0, 100.0	100.0	84.54, 100.0

EBF -Exclusive Breast Feeding

## Discussion

The present study showed that in the study population, both maternal recall method data as well as data retrospectively obtained using an event calendar method systematically overestimates the duration of EBF. Maternal recall data does not seem to be valid at all in estimating the duration of EBF. Use of an event calendar provided much more valid data.

Several previous studies also showed disagreement between data since birth and recall data. However, most of those studies reported better agreement than the present study. Very few studies have specifically studied the time of introduction of water or other liquids which the present study collected data as the date when EBF ended [11]. Bland et al in their study showed that at nine months, specificity of reported EBF ranged from 40 to 82%. In their study the sensitivity was high, ranging around 80%. However, previous studies have shown that when the recall period was longer the misclassification of EBF period was also large. For shorter recall periods, it has been shown that maternal recall data are comparable with data since birth. The present study found low validity of respondent reported data in determining EBF retrospectively. A reason for this difference could be the way the questions were framed in maternal recall. Asking the duration of EBF (as in this study) will provide different results to asking the time of onset of complementary feeding.

Recall bias is a well-known phenomenon in epidemiological studies. However, the low validity found in this study cannot be directly attributed to recall bias. The reported data depends mainly on formulating of appropriate questionnaires and methods. In a country like Sri Lanka with high literacy rate, especially among females (89.7%) [13], where people are aware of the recommended duration of EBF, mothers tend to provide answers that reflect the desired duration rather than what are practised. This social desirability bias could have affected the present study, because data collectors were their service providers. The main reason for obtaining acceptable results in the event calendar method may be due to the authors not mentioning EBF during the particular assessment, making mothers not obligatory to report the actual situation. However, in large population based studies, event calendar method could be difficult to administer with large number of other variables. At least a list of common supplementary foods including water and other juices could be used to probe into the duration of EBF.

There were several limitations in the present study. This sample was not a probability sample and the generalization of results is limited. Data collection was carried out by the service providers who knew about the study hypothesis, which could lead to probable interviewer bias. Wording of recall questionnaire "at what age you discontinued EBF?" could provide different results than the other popular method of asking "at what age you started complementary feeding". However, within the given limitations results of this study could be used to improve the quality of data in studies where breastfeeding is a major determinant. We propose reporting data accrual methods in breastfeeding studies as an essential requirement in its' methods section. Use of event calendars rather than direct questioning is recommended to evaluate the EBF duration.

## Conclusions

Retrospective evaluation methods systematically overestimate the duration of EBF. Reporting of data accrual methods in breastfeeding studies will allow the readers to interpret finding accurately and the use of event calendars rather than direct questioning as a valid method of determining EBF is recommended.

This study was partially funded by Rajarata University Research Grant 2009 (Grant No-RJT/R&P/2009/Med/R.03)

## Competing interests

The authors declare that they have no competing interests.

## Authors' contributions

ASB designed the study, analyzed and interpreted data, and prepared the manuscript.

SF involved in the design, data collection and manuscript preparation. DSD and TCA helped in analysis and interpretation of data and in the preparation of the manuscript.

All authors read and approved the manuscript.

## Pre-publication history

The pre-publication history for this paper can be accessed here:

http://www.biomedcentral.com/1471-2431/11/80/prepub
